# Phase slips extracted from derivatives of EEG data provide a deeper insight into the formation of cortical phase transitions

**DOI:** 10.3389/fnint.2025.1471120

**Published:** 2025-02-25

**Authors:** Ceon Ramon, Paolo Gargiulo

**Affiliations:** ^1^Department of Electrical and Computer Engineering, University of Washington, Seattle, WA, United States; ^2^Regional Epilepsy Center, University of Washington, Seattle, WA, United States; ^3^Department of Engineering, Institute of Biomedical and Neural Engineering, Reykjavik University, Reykjavik, Iceland; ^4^Department of Science, Landspitali University Hospital, Reykjavik, Iceland

**Keywords:** EEG phase slips, cortical phase transitions, high-density EEG, EEG differentiation, EEG derivatives

## Abstract

The phase slips are generally extracted from the EEG using Hilbert transforms but could also be extracted from the derivatives of EEG, providing additional information about the formation of cortical phase transitions. We examined this from the 30 s long, 256-channel resting state, eyes open EEG data of a 30-year-old male subject. The phase slip rates, PSR1 from EEG, PSR2 from the first-order derivative of EEG, and PSR3 from the second-order derivative of EEG, respectively, were extracted. The study was performed in the alpha (7–12 Hz) band only. The spatiotemporal plots of the EEG and phase slip rates over a 3.0 s period with a 0.5 s resolution were made with a montage layout of the 256 electrode positions. The spatiotemporal patterns of EEG and its derivatives exhibited shifting activity from posterior visual areas to the central and frontal regions over the 3.0 s period. The PSR1, PSR2, and PSR3 activity areas were different from the EEG and were distributed in larger areas as compared with the EEG and its derivatives. Also, the PSR2 and PSR3 activity areas and magnitudes were significantly different as compared with the PSR1 alone. This was also confirmed (*p* < 0.01) by the one-way ANOVA analysis of the means of PSR1, PSR2, and PSR3. These results show that PSR2 and PSR3 carry additional information that could potentially be biomarkers for studying the rate of formation of phase slips and the related cortical activity from the derivatives of EEG data.

## 1 Introduction

The coordinated activity of a group of neurons at mesoscopic and sub-mesoscopic scales (~0.1–05 mm) is always in a state of criticality (Freeman et al., 2003; Kozma and Freeman, 2017). Any slightest input, e.g., a visual stimulus or a thought, could trigger a phase transition, leading to small perturbations in the EEG data. These produce sharp episodic phase slips in the detrended unwrapped phase of the EEG, which can be extracted using Hilbert transform techniques (Ramon et al., 2023, 2024). These phase slips refer to the transition of neuronal activity between synchronous and asynchronous states, representing cortical phase transitions. Theoretical models of these cortical phase transitions are very similar to the Ising model of ferromagnetism (Beggs and Timme, 2012). A recent review paper summarizes many other examples of criticality and phase transitions in physics and biology (Heffern et al., 2021).

Typically, the phase slips are extracted from EEG. However, these can also be extracted from the first (*d/dt*) and second (*d^2^/dt^2^*) order derivatives of the EEG, giving us additional information about how fast the cortical phase transitions are happening. This is the main theme of this study. The phase slips derived from the EEG and their derivatives provide unique opportunities to study the behavior of the cortical phase transitions. We have applied these new tools to study the cortical phase transitions from the resting state EEG in the alpha (7–12 Hz) band because its power is very high, and its phase-modulated activity changes during tasks (Ramon et al., 2023), the transition from wakefulness to drowsiness (Kalauzi et al., 2015), brain stimulation (Aksenov et al., 2024), etc. However, these tools are generic and could be applied to any EEG data and in different EEG bands. The time-varying changes in EEG are related to the dynamic changes in the neuronal population at a mesoscopic scale that acts in a synchronized fashion (Pinotsis et al., 2012). Thus, the phase slip rates extracted from the EEG derivatives might help us better understand the underlying cortical neurodynamics and formation of burst oscillations. This understanding could have significant implications for resting-state brain behavior, event-related potentials, seizure evolutions, and many other aspects of brain function (Freeman and Quiroga, 2013). However, these need to be investigated with carefully designed studies.

In general, the first and second-order differencing of the time series EEG data is not a common procedure in EEG data analysis. In the past, it has been used to identify and eliminate the false ripples in the EEG data (Al-Bakri et al., 2022; Kobayashi et al., 2021), tracking of alpha band activity during anesthesia (Obert et al., 2022), and to analyze self-organized criticality and the phase reset (Thatcher et al., 2009, 2014). However, the application of derivatives of EEG to study the cortical phase transitions is relatively a new procedure, to our knowledge. The first and second-order differentiations act like high pass filters which eliminate the linear trends and enhance the high-frequency signals in a given time series data set. The differentiation also causes the slope reversal which helps in better identification of the location of peaks in a time-series data (Mukhopadhyay et al., 2012; O’Haver, 2023). Similarly, the application of the Hilbert transform introduces a ± π, i.e., a total of 2π phase shift at the locations of changes in the slope and perturbations in the given time series data. This should be noted that the ±π phase shifts are not related to the amplitude of EEG or ECoG (Ramon et al., 2024; Ramon et al., 2023). Therefore, in summary, it is a possibility that a combination of differentiation and Hilbert transform might help to extract phase slips related to tiny perturbations in the EEG data which might be buried in the background random noise. Of course, one has to apply biological constraints to separate the phase slips due to biological processes as compared with the phase slips from the random noise (Ramon et al., 2023). Our reported results indicate that this is what is happening and this possibly could give us additional biomarkers to study the formation of cortical phase transitions from the derivatives of EEG data. Just as a note, in our previous work (Ramon et al., 2024), we took the derivatives of PSR by differentiating them. In contrast, in the current work, the PSR is computed from the derivatives of EEG, which relate directly to the changes in the neuronal populations.

In the following sections, we give details of our methods, results, discussion, and conclusions.

## 2 Materials and methods

### 2.1 EEG data and phase slip rate computations

**Figure 1:**
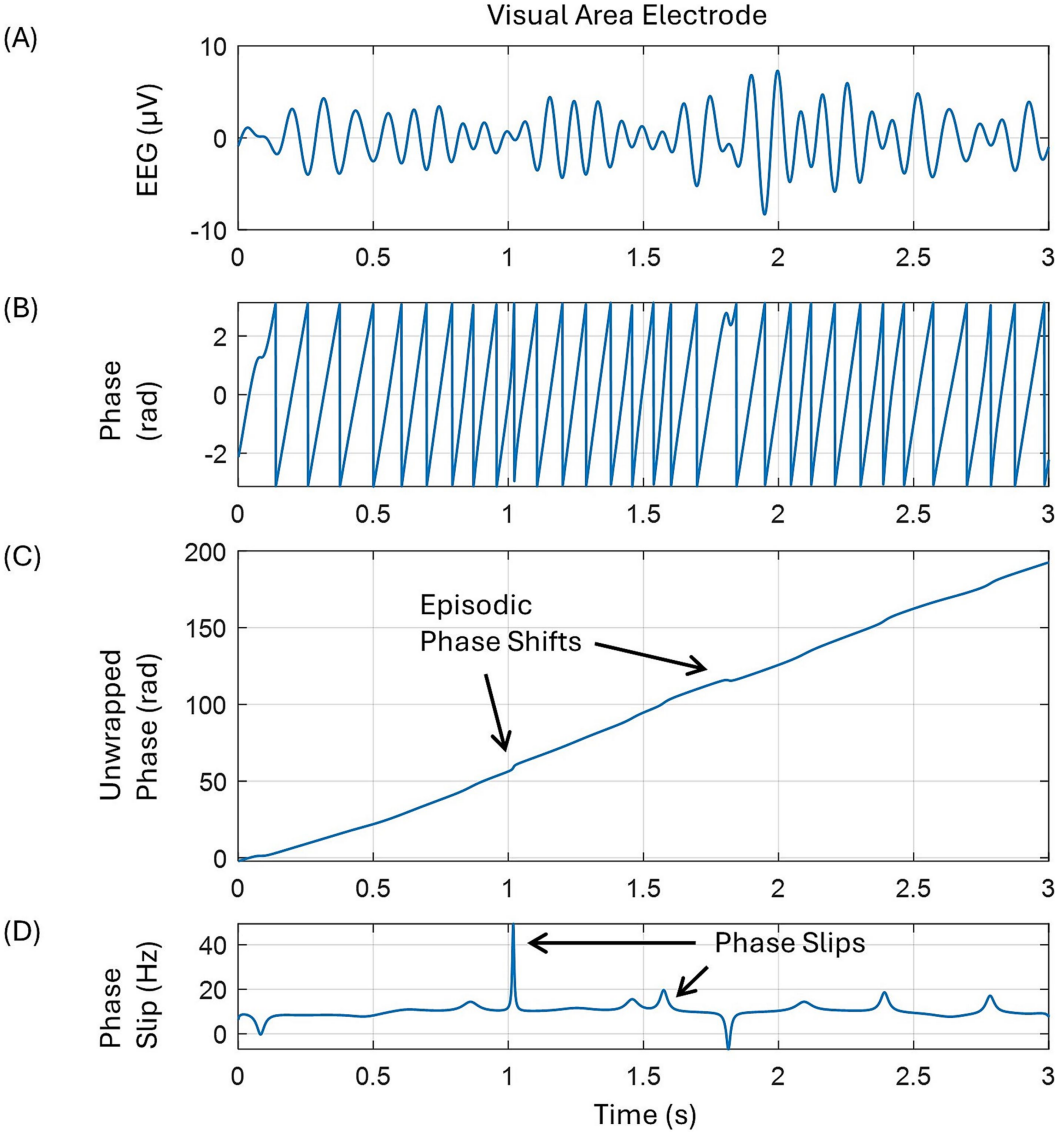
A pictorial representation of the mathematical procedures to extract phase slips from the EEG data. **(A)** EEG trace in the alpha band from one of the electrodes in the left occipital area, back of the head, **(B)** saw tooth pattern of the spontaneous phase after taking Hilbert transform of the EEG, **(C)** unwrapped phase showing some episodic phase shifts, and **(D)** phase slips after taking the derivative of the unwrapped phase.

The phase slip rates (PSR) were extracted from the EEG and the EEG data’s first and second-order derivatives of the 256-channel resting state, eyes open EEG data of a 30-year-old male subject. The data was collected with an ANT Neuro 256-channel system at Reykjavik University, Iceland, under Iceland’s approved human subjects guidelines. The data was originally collected at a 16,384 Hz sample rate for 5 min. Randomly selected data of continuous 30 s duration was imported into MATLAB and down-sampled to 1,024 Hz for further analysis. The data was filtered with an equiripple filter in a broad band of 3–49 Hz and then re-referenced to the common averaged reference. By use of the ICA (independent component analysis) techniques, the muscle, eyeblink, and heartbeat artifacts were removed. For this, we used the EEGLAB software. This cleaned-out EEG data was then filtered in the alpha (7–12 Hz) band with an equiripple filter and then used for phase slip extraction and PSR computations. The sawtooth patterns of the phase were extracted from the EEG data using Hilbert transform, which on unwrapping and detrending shows phase slips at the location of small perturbations in EEG data related to cortical phase transitions. A pictorial representation of these mathematical procedures is given in Figure 1. Some large phase slips are marked. These were obtained by taking the derivative of the unwrapped phase, which will be in the units of rad/s. Dividing this by 2π gives us the phase frequency in cycles/s or Hz.

The PSR from the phase slips was calculated in a stepping window of 10 ms duration with a step size of one digitization point which is equal to 1/1,024 s = 0.97 ms. These procedures were repeated for the EEG data’s first and second-order derivatives also. EEG and PSR spatial plots were constructed with a montage layout of 256 electrode positions [(Ramon et al., 2023); Refer to Figure 3] on a flat surface that included the approximate location of some prominent functional areas on the scalp surface (Dixon et al., 2017; Gazzaniga and Mangun, 2014).

To separate the phase slips arising from the biological processes versus the random noise, several criteria were applied. These include: (1) phase slip frequency is within the alpha band of 7–12 Hz, (2) sign of the positive or negative peaks did not change for at least three consecutive time steps, and (3) the magnitude of the three consecutive peaks was within the mean ± 1.05σ of the three peaks. Application of these criteria helped to significantly reduce the counting of phase slips due to random noise and at the same time maximize the counting of phase slips from the biological processes. The differentiation of the EEG signal reduces the low-frequency signal while amplifying the high-frequency signal and the background noise (Chaddad et al., 2023; Mensen et al., 2017). This could potentially introduce an error in phase slip computations from the derivatives of the EEG signal. However, it is important to note that these biological constraints still help to reduce the erroneous counting of phase slips from the amplified random noise. The next section will provide further insights into this. The reason is that the amplified random noise will likely have different slopes for consecutive time steps, and their amplitudes might be out of bounds for a given frequency band.

### 2.2 Surrogate data testing

After filtering in the alpha band, the PSR of random noise was computed from the randomly shuffled EEG data. We used the ‘randperm’ command in the MATLAB software for this. These techniques have been used before by us (Ramon et al., 2024; Ramon et al., 2023) and also described in a recent paper (Lancaster et al., 2018) on surrogate data analysis. The power spectral density of the randomly shuffled EEG data was significantly different from the original EEG data, and it was close to a white noise. The phase slips were extracted from the randomized EEG data and then randomly shuffled. After that, the PSR was computed in a stepping window of 10 ms duration with a step size of one digitization point. The mean and standard deviations of PSR were found to be zero after averaging over *n* = 100 trials. A similar analysis was also performed on the PSR derived from the first and second-order derivatives of the randomly shuffled EEG data. It was found that in these cases, the mean and standard deviations of PSR from the shuffled data were also zero after averaging over *n* = 100 trials. This shows that our reported results are from the biological processes extracted from the EEG data above the random noise.

### 2.3 Computational resources

The data analysis was performed on a desktop computer with an 8-core CPU and 112 GB of memory using EEGLAB (Delorme and Makeig, 2004). For computations of PSR requiring more significant (>112 GB) memory, we used MATLAB software at the UCSD (University of California, San Diego) supercomputing center using their NSG (Neuroscience Gateway) portal.

## 3 Results

### 3.1 Temporal plots of one electrode

**Figure 2:**
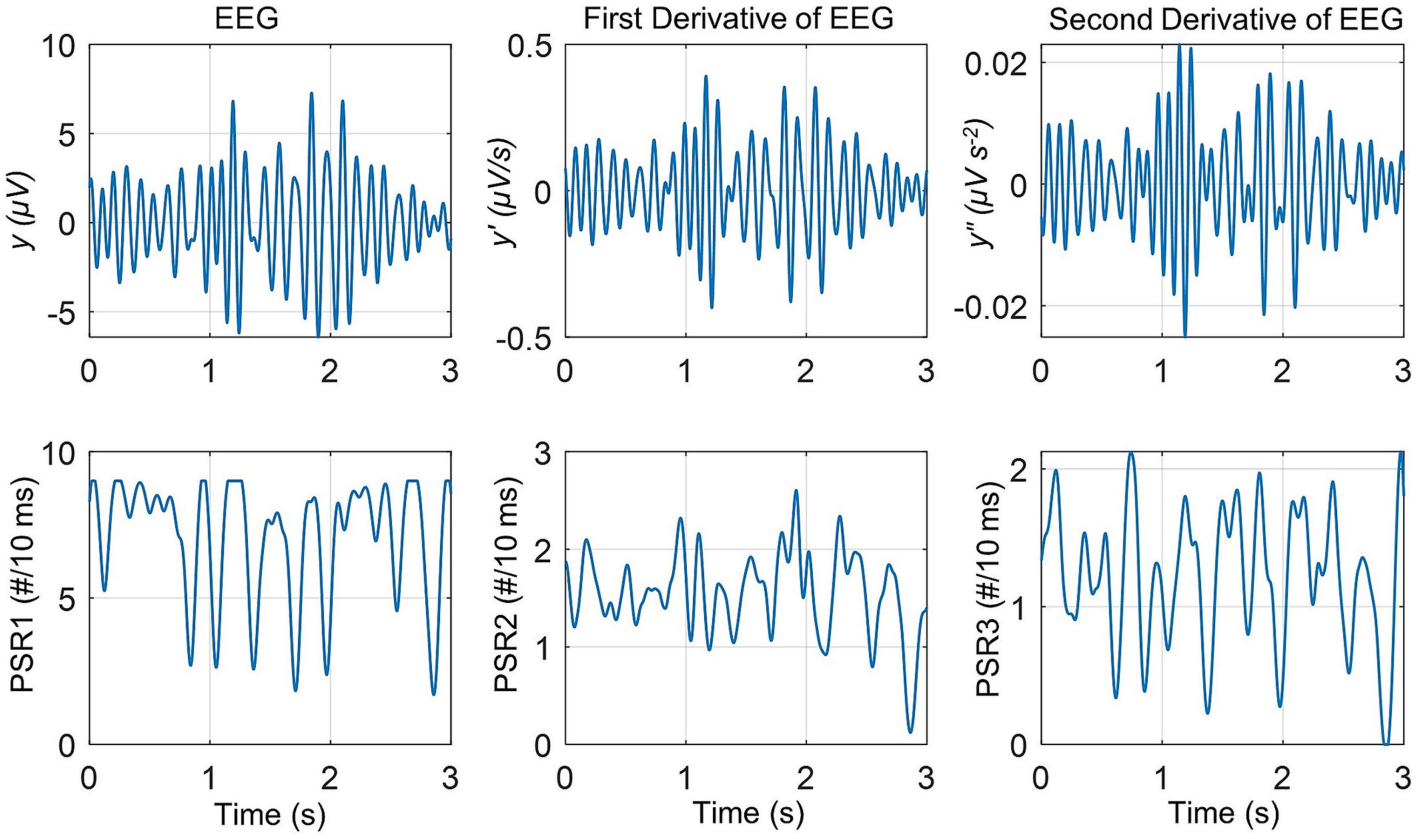
The EEG and its first and second derivatives are in the top row, followed by the phase slip rates in the bottom row. The PSR1 is the phase slip rate extracted from the EEG, *y*. The PSR2 is the phase slip rate extracted from the first-order derivative of EEG, *y′*. The PSR3 is the phase slip rate extracted from the second-order derivative of EEG, *y″*.

The EEG, its derivatives, and the PSR computed from these for one of the electrodes in the front central area are given in Figure 2. The top row is for the EEG voltage, *y* (μV), first-order derivative, *y′* (μV/s), and the second-order derivative, *y″* (μV s^−2^). The bottom row is for the PSR derived from the EEG and its derivatives. Here EEG voltage is represented as a time series data: *y = y_1_* + *y_2_* + … + *y_n_*, as used earlier (Kobayashi et al., 2021). The PSR1 is from the EEG, the PSR2 is from the first derivative of EEG, and the PSR3 is from the second derivative of EEG, respectively.

The variable, *y*, is the EEG filtered in the alpha (7–12 Hz) band with sinusoidal amplitude varying between ±7 μV. The first-order derivative, *y′* (μV/s), represents the rate of change of the EEG signal and its amplitude varies between ±0.4 μV/s. The second derivative, *y″*, is the acceleration of the EEG signal, *y*, and its amplitude varies between ±0.02 μV s^−2^. One can also see the slow spindling oscillations of theta (3–7 Hz) band over the carrier frequency of the alpha (7–12 Hz) band. These are observable in the EEG signal *y*, and also in the derivatives, *y′*, and *y″*.

The magnitude of the PSR1 varies between 1.7 and 9.0 counts/(10 ms), or #/(10 ms). Here # refers to the number of counts. The magnitude of the PSR2 varies between 0.12 and 2.6 #/10 ms. The magnitude of the PSR3 varies between 0.0 and 2.12 #/10 ms.

As anticipated, taking the derivatives of EEG removes the linear trends in the EEG data and slightly amplifies the high-frequency perturbations in the EEG data. The one-way ANOVA (analysis of variance) analysis was performed on the PSR1, PSR2, and PSR3. It was found that the means of all three quantities were significantly (*p* < 0.01) different from each other. A similar ANOVA analysis was performed on the PSR of all other 255 electrodes. It was found that for each electrode, the means of PSR1, PSR2, and PSR3 were significantly (*p* < 0.01) different from each other.

### 3.2 Spatial plots

**Figure 3:**
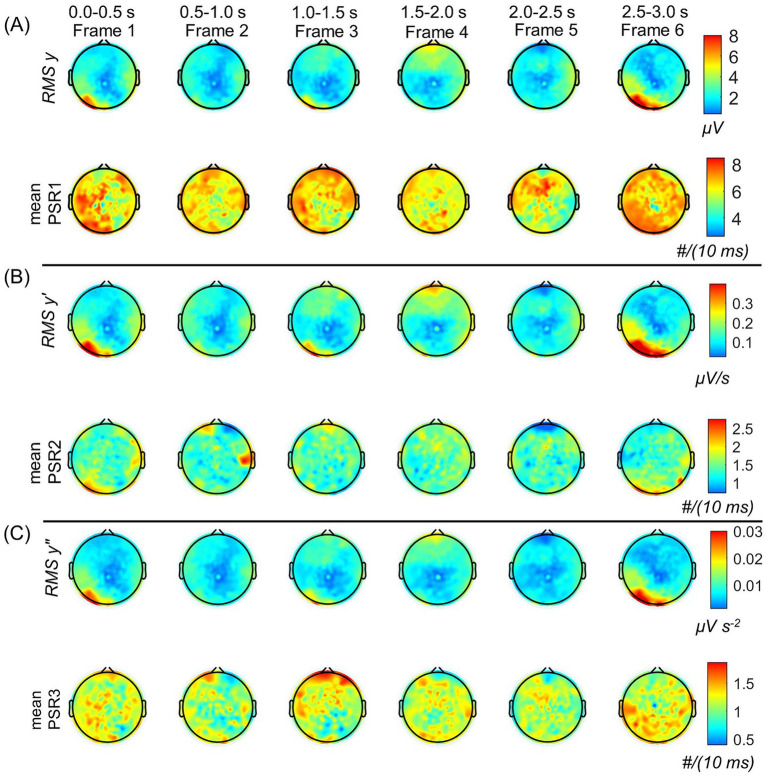
Spatiotemporal plots of the root mean square (RMS) values of EEG and its derivatives, and their associated mean values of phase slip rates. The RMS and mean values were computed in the stepping window of 0.5 s duration. The temporal duration for each frame is given at the top. **(A)** The top two rows are for the RMS *y* (μV) and the mean PSR1 (#/(10 ms)), respectively. **(B)** The middle two rows are the for the RMS *y′* (μV/s) and the mean PSR2 (#/(10 ms)), respectively. **(C)** The bottom two rows are the RMS *y″* (μV s^−2^) and the mean PSR3 (#/(10 ms)), respectively. Notice the spatial changes in going from one frame to the next.

For the spatial plots, we have computed the root mean square (RMS) values for the EEG and its derivatives, i.e., *y*, *y′*, and *y″*. For a sinusoidal time-varying signal, it is better to use RMS values as compared with the mean of the signal. The RMS values were computed within a stepping window of 0.5 s covering the period of 0.0–3.0 s. This was an arbitrary choice to show the changes in six frames of spatiotemporal patterns during the range of 3.0 s as shown in Figure 3. However, a shorter window could also be used for finer details.

Figure 3 is divided into three sections, (A), (B), and (C). The top two rows in section (A) refer to the spatial plots of RMS EEG, i.e., RMS *y* (μV), and the mean values of associated phase slip rate, i.e., mean of PSR1 (#/(10 ms)), respectively. The RMS and mean values were computed in the stepping window of 0.5 s duration. Similarly, the middle two rows marked as section (B) are the spatial plots of the RMS value of the first-order derivative of EEG, i.e., RMS *y′* (μV/s), and the mean values of its associated phase slip rate, PSR2 (#/(10 ms)), respectively. The bottom two rows marked as section (C) are the spatial plots of the RMS values of the second-order derivative of EEG, i.e., RMS *y″* (μV s^−2^), and the mean PSR3 (#/(10 ms)), respectively. The temporal duration for each frame and the frame numbers are given at the top.

Looking at Figure 3A, the focus of the spatial location of RMS *y* shifts from the left visual (posterior left) in Frame 1 to the front central area in Frame 4 and then again to the left visual area in Frame 6. This would suggest that during the 3.0 s period, the brain activity shifts from the left visual to the front central and then to the left visual area again. Another point to note is that in the majority of the frames, the midline central areas have very low activity. In comparison to *y*, i.e., RMS EEG potentials, the mean values of the PSR1 are spread in wide areas in each frame. In addition, there are some noticeable spatial differences from one frame to the next frame. In Frame 1, most activity is on the left side visual, posterior, and central areas. Then in Frame 2 and Frame 3, it shifts slightly in the frontal and the right front central areas. The activity is reduced in magnitude all over in Frame 4, but the spatial pattern is similar to Frame 3. After that in Frame 5, it is mainly concentrated in the left temporal and frontal areas. It is widespread all over in Frame 6 with maximum activity in the left visual area which matches with the activity area of RMS *y*. Similar spatiotemporal differences between EEG activity and the PSR have been observed earlier in a visual evoked potential study (Ramon et al., 2023).

A one-way ANOVA analysis was performed on the six frames of RMS *y* and it was found that they were significantly (*p* < 0.01) different from each other. Similarly, a one-way ANOVA analysis was also performed on the six frames of the mean PRS1 and it was found that they were significantly (*p* < 0.01) different from each other. Overall the spatial patterns of the mean PSR1 activity are significantly different from the RMS *y* activity. This was confirmed by the paired t-test for each frame between the two variables, RMS *y* and mean PSR1. It was found that they were significantly (*p* < 0.01) different for each of the six frames.

In Figure 3B, the spatial plots for six frames for the RMS *y′* and the mean of PSR2 are given. These spatial plots of RMS *y* (Figure 3A) and RMS *y′* (Figure 3B) are very similar. This would suggest that the EEG and its first derivatives are spatially in the same place, i.e., at the same electrodes. In comparison, the spatial plots of the means of PSR1 and PSR2 are very different. In Frame 1, the mean PSR1 activity is spread on the left side in a wide area, while the mean PSR2 activity is strongly focused in the left visual area. Similar differences are observable in the other frames also. One thing to note is that the spatial patterns of RMS *y′* and the PSR2 are very similar with heightened activity in the visual and the frontal regions in most of the frames. A one-way ANOVA analysis was performed on the six frames of RMS *y′* and it was found that they were significantly (*p* < 0.01) different from each other. Similarly, a one-way ANOVA analysis was also performed on the six frames of the mean PRS2 and it was found that they were significantly (*p* < 0.01) different from each other. The paired t-test for each frame between the two variables, RMS *y′*, and mean PSR2 was performed. It was found that they were significantly (*p* < 0.01) different from each other for all of the six frames.

The spatial plots of RMS *y″* and the mean PSR3 are given in Figure 3C. The spatial patterns of RMS *y*, RMS *y′*, and RMS *y″* are very similar. However, spatial patterns of the mean PSR2 and mean PRS3 are very different in each of the six frames. There are some similarities between the spatial plots of the mean PRS1 and the mean PRS3. A one-way ANOVA analysis was performed on the six frames of RMS *y″* and it was found that they were significantly (*p* < 0.01) different from each other. Similarly, a one-way ANOVA analysis was also performed on the six frames of the mean PRS3 and it was found that they were significantly (*p* < 0.01) different from each other. The paired t-test for each frame between the two variables, RMS *y″*, and the mean PSR3 was performed. It was found that they were significantly (*p* < 0.01) different from each other for all of the six frames.

### 3.3 Results from other data segments

Similar analyses as given in Figure 3 were performed on 10 randomly selected EEG data of 3.0 s duration out of 30 s long data. For each trial RMS values of *y*, *y′*, and *y″* over a 3.0 s duration were computed. Also, the mean values over a 3.0 s period for PSR1, PSR2, and PSR3 were computed. This gives us 256 electrode values for each variable, i.e., RMS *y*, RMS *y′*, etc. A one-way ANOVA analysis was performed on the RMS values of *y*, *y′*, and *y″*, and also on the mean values PSR1, PSR2, and PSR3. It was found that the RMS values of *y*, *y′*, and *y″* were significantly (*p* < 0.01) different from each other. Similarly, the mean values PSR1, PSR2, and PSR3 were also significantly (*p* < 0.01) different from each other.

Later on, the quantities were averaged over all 256 electrodes and are written as: $\bar{y}$ , $\bar{y^{\prime }}$ , $\bar{y^{\prime \prime }}$ , $\bar{PSR1}$ , $\bar{PSR2}$ , and $\bar{PSR3}$ . To get $\bar{y}$ , first, the RMS value of the EEG potentials for each electrode was computed for the time period of 3.0 s and then averaged over the values for all 256 electrodes. For the computation of $\bar{PSR1}$ , first, the mean value of the PSR1 for each electrode was computed for the time period of 3.0 s and then averaged over the values for all 256 electrodes. Similar procedures were used for the remaining quantities and all are tabulated in Table 1.

**Table 1:** Averaged values for each trial.

Trial number	$\bar{y}$ (μV)	$\bar{y^{\prime }}$ (μV/s)	$\bar{y^{\prime \prime }}$ (μV s^−2^)	$\bar{PSR1}$ #/(10 ms)	$\bar{PSR2}$ #/(10 ms)	$\bar{PSR3}$ #/(10 ms)
1	3.16	0.20	0.0129	4.20	1.03	1.20
2	2.83	0.17	0.0108	5.12	1.25	1.20
3	2.56	0.15	0.0096	5.53	1.34	1.21
4	2.39	0.14	0.0085	5.82	1.38	1.21
5	2.35	0.14	0.0085	5.95	1.38	1.16
6	2.51	0.15	0.0090	6.15	1.41	1.16
7	3.33	0.19	0.0120	6.33	1.51	1.21
8	3.49	0.20	0.0122	6.61	1.57	1.29
9	3.45	0.20	0.0120	6.51	1.55	1.29
10	2.72	0.15	0.0091	6.44	1.46	1.27
Mean ± std	2.88 ± 0.44	0.17 ± 0.03	(10.5 ± 2) × 10^−3^	5.87 ± 0.75	1.39 ± 0.16	1.22 ± 0.05

First, the RMS values of EEG, *y*, and its derivatives, *y′*, and *y″* over a 3.0 s period were computed and then averaged over all 256 electrode values. These quantities are: $\bar{y}$ , $\bar{y^{\prime }}$ , and $\bar{y^{\prime \prime }}$ . For the phase slip rates, *PSR1*, *PSR2*, and *PSR3*, the mean values over a 3.0 period were computed and then averaged over all 256 electrode values. These quantities are: $\bar{PSR1}$ , $\bar{PSR2}$ , and $\bar{PSR3}$ .

A one-way ANOVA analysis with two groups was performed. One group was: $\bar{y}$ , $\bar{y^{\prime }}$ , and $\bar{y^{\prime \prime }}$ . The other group was: $\bar{PSR1}$ , $\bar{PSR2}$ , and $\bar{PSR3}$ . It was found that values for each group were significantly different from each other (*p* < 0.01). This statistical analysis shows that the information contained in PSR1, PSR2, and PSR3 is significantly (*p* < 0.01) different from each other and will suggest that one can derive additional information from the first and second-order derivatives of EEG data.

## 4 Discussion

Our results indicate that PSR obtained from the first and second derivatives of the EEG data have additional information as compared with the PSR derived from the EEG data alone. This was the main objective of this investigation. This additional information might be related to how fast the phase slips are arising which in turn relates to how fast and how many neurons are taking part in the cortical phase transitions. In general, coordinated activity of neurons happens at the minicolumn level which is about 28–40 μm in size and contains about 80–120 neurons (Bennett, 2020; Mountcastle, 1957; Sporns et al., 2005). These minicolumns are also called microcolumns in the literature. Neurons in these minicolumns are interconnected, have common inputs and outputs, and form basic computational units for the genesis of burst oscillations and subsequent local field potentials in the cortex and then the scalp EEG potentials (Friston et al., 2015; Pinotsis et al., 2012). The coordinated activity of neurons at minicolumn resolution could also be considered as a building block of cortical phase transitions (Freeman and Vitiello, 2006, 2010; Freeman and Quiroga, 2013) which give rise to the phase slips in the EEG data. Thus, in a sequential causative fashion, the phase slips and the PSR derived from the EEG and the derivatives of EEG give us additional information about the cortical phase transitions. However, all of this needs to be ascertained through large-scale simulation studies which we hope some research groups will take on this task. Another point to note is that, in general, the coordination of the neuronal activity happens at the minicolumn level. However, minicolumns in different parts of the brain are connected through axonal pathways. Thus, one could see this coordination of neuronal activity at the local and global levels in the whole cortex and this is what generally is observed in phase synchronization and phase slip studies from the EEG and ECoG data (Freeman et al., 2003; Ramon and Holmes, 2015; Ruiz et al., 2010).

The phase slip rate, PSR1 is extracted from the EEG data and tells us how many cortical phase transitions are happening in a given time window. Here we have used a stepping time window of 10 ms which gives us PSR1 in the range of 0–10 counts/(10 ms). The PSR2 is extracted from the first derivative (*d/dt*) of the EEG, *y′* (μV/s). The PSR2 is related to the rate of change of EEG and it is not the derivative of PSR1. The rate of change of EEG, i.e., *y′*, would depend on how many neurons at any given moment are joining or leaving the process of cortical phase transitions. In a simple way, the PSR2 represents the rate of change of EEG which in turn relates to the rate of change of cortical phase transitions, and which in turn relates to the changes in the population of neurons taking part in the process of cortical phase transitions (Freeman and Vitiello, 2006, 2010). Similarly, the PSR3 will represent the acceleration, or deceleration, of the EEG which in turn will relate to the acceleration or deceleration of cortical phase transitions.

The spatial plots for PSR in the alpha band in our previous work [(Ramon et al., 2023), Figure 6] are significantly different as compared with PSR1 spatial plots (Figure 3A) of the current work. This difference, stemming from the previous study’s focus on visual object naming tasks and the current research on the resting state eyes open, could potentially be a reason. The electrical activity related to the default mode networks for the resting state is spread in the broader brain area that is deactivated and replaced with focused brain areas during cognitive tasks (Menon, 2023; Zhang et al., 2022). This needs further investigation with a properly designed study.

Our main objective in this study was to develop a new technology for computing PSR from the derivatives of EEG and show its efficacy in studying cortical phase transitions. Our results have reliably shown this from one subject in the alpha (7–12 Hz) band. These findings should be further studied in the different EEG bands, such as in theta (3–7 Hz), beta (12–30 Hz), low gamma (30–50 Hz) and high gamma (50–80 Hz) bands. Further extension to a higher frequency (80–250 Hz) band might also be good which often are characterized by epileptogenic activities (Kobayashi et al., 2021; Ramon and Holmes, 2020; Ramon et al., 2018). Similarly, the application of these procedures to other types of EEG data sets will also be helpful.

Overall, we summarize that the PSR derived from the first and second-order derivatives of EEG provide additional information to the PSR derived from the EEG alone. In this respect, this technique could be considered as an additional biomarker to the traditional methods, such as EEG power or phase-amplitude coupling, to study the brain behavior from the EEG data.

## Author’s note

Some preliminary results were presented as a virtual poster at the 10th Annual BRAIN Initiative Conference: celebrating a decade of innovation. June 17–18, 2024; Rockville, MD, USA and Virtual. Poster #349. Poster title: EEG derivative-related phase slips provide a deeper insight into cortical phase transitions.

## Data Availability

Publicly available datasets were analyzed in this study. This data can be found at: https://osf.io.
